# Identification of Dysregulated microRNA Networks in Schwann Cell-Like Cultures Exposed to Immune Challenge: Potential Crosstalk with the Protective VIP/PACAP Neuropeptide System

**DOI:** 10.3390/ijms19040981

**Published:** 2018-03-25

**Authors:** Giuseppe Musumeci, Gian Marco Leggio, Rubina Marzagalli, Ghaith Al-Badri, Filippo Drago, Alessandro Castorina

**Affiliations:** 1 Section of Human Anatomy and Histology, Department of Biomedical and Biotechnological Sciences, University of Catania, via S. Sofia, 87, 95123 Catania, Italy; giumusu@unict.it (G.M.); rubinamarzagalli@yahoo.it (R.M.); 2 Section of Pharmacology, Department of Biomedical and Biotechnological Sciences, “Torre Biologica”, University of Catania, via S. Sofia, 97, 95123 Catania, Italy; gmleggio@me.com (G.M.L.); fdrago@unict.it (F.D.); 3 School of Life Sciences, Faculty of Science, University of Technology Sydney, P.O. Box 123, Broadway, Sydney NSW 2007, Australia; gaith.al-badri@uts.edu.au; 4 Discipline of Anatomy and Histology, School of Medical Sciences, the University of Sydney, Sydney NSW 2006, Australia

**Keywords:** miRNA, PACAP, VIP, Schwann cells, peripheral nerve, lipopolysaccharide, inflammation, neuropeptides

## Abstract

Following peripheral nerve injury, dysregulations of certain non-coding microRNAs (miRNAs) occur in Schwann cells. Whether these alterations are the result of local inflammation and/or correlate with perturbations in the expression profile of the protective vasoactive intestinal peptide (VIP)/pituitary adenylate cyclase-activating polypeptide (PACAP) system is currently unknown. To address these issues, we aimed at profiling the expression of selected miRNAs in the rat RT4 Schwann cell line. Cells exposed to lipopolysaccharide (LPS), to mimic the local inflammatory milieu, were appraised by real-time qPCR, Western blot and ELISAs. We found that upon LPS treatment, levels of pro-inflammatory cytokines (IL-1β, -6, -18, -17A, MCP-1 and TNFα) increased in a time-dependent manner. Unexpectedly, the expression levels of VIP and PACAP were also increased. Conversely, levels of VPAC1 and VPAC2 receptors were reduced. Downregulated miRNAs included *miR-181b*, *-145*, *-27a*, *-340* and *-132* whereas upregulated ones were *miR-21*, *-206*, *-146a*, *-34a*, *-155*, *-204* and *-29a*, respectively. Regression analyses revealed that a subset of the identified miRNAs inversely correlated with the expression of VPAC1 and VPAC2 receptors. In conclusion, these findings identified a novel subset of miRNAs that are dysregulated by immune challenge whose activities might elicit a regulatory function on the VIP/PACAP system.

## 1. Introduction

Axons of the peripheral nervous system (PNS), as opposed to those of the central nervous system (CNS), exhibit high capacity of regeneration after lesion. This is due to intrinsic properties of PNS neurons, but also largely depends on the activity of extrinsic factors that allow and promote axonal regeneration in the PNS [1]. Schwann cells (SCs), the PNS myelinating glia, hold major functions in creating an environment that is favorable for axonal regrowth, stimulating axon outgrowth after lesion, and rebuilding myelin sheaths of regenerated axons [2]. In intact nerves, SCs are present in two differentiated states, either myelinating axons (myelinating SCs) or ensheathing groups of small-calibre axons in Remak bundles (non-myelinating SCs). In contrast, following injury, differentiated SCs reprogram to progenitor-like cells. Dedifferentiated SCs shut down the myelination program and acquire new phenotypes coordinately to support nerve repair. These phenotypes include (1) secretion of neurotrophic factors to promote axonal survival; (2) clearance of myelin debris to generate a favorable environment for axonal regrowth; (3) initiation of inflammatory responses to promote wound healing; and (4) proliferation to replace dead cells [1,3].

In previous studies, we have demonstrated that two naturally occurring neuroprotective peptides, pituitary adenylate cyclase-activating polypeptide (PACAP) and the structurally related vasoactive intestinal peptide (VIP), are actively involved in regulating some of the above indicated phenotypic changes in SC lines [4,5,6]. The biological functions of these peptides are mediated by two receptor subtypes, PAC1 and VPAC receptors. PAC1 has very high affinity for PACAP and lower for VIP, whereas VPAC receptor subtypes (comprising VPAC1 and VPAC2 receptors) display equal and high affinity for both PACAP and VIP [7,8,9]. Due to the intrinsic complexity and number of functions elicited by these peptides in SCs, we reasoned that PACAP and VIP biological activities could be the result of a crosstalk between the endogenous VIP/PACAPergic system and a system capable of epigenetically controlling the expression of multiple genes. Interestingly, recent evidence has proposed that following peripheral nerve injury, the progressive transition of SCs from a myelinating phenotype towards the different progenitor-like phenotypes is largely dependent on the wound microenvironment, which features the local presence of several growth factors and a robust acute inflammatory activity [10]. According to these authors, some elements of the wound microenvironment may trigger post-transcriptional changes to initiate in SCs the reprogramming that ultimately supports the peripheral nerve regeneration process [10]. In this context, we questioned whether a class of small non-coding molecules, namely microRNAs (miRNAs), could play a role in SCs reprogramming in light of the fact that miRNAs are potent post-transcriptional regulators able to regulate the expression of up to 1000 genes [11].

MicroRNAs (miRNAs) are small (approx. 22 nucleotides) non-coding RNAs capable of post-transcriptionally silencing mRNAs that contain sequences complementary to the miRNAs’ 7- to 8-bp “seed” sequence [12,13]. MiRNAs have been implicated as regulators of various cellular and physiological processes such as differentiation, proliferation, and cancer (reviewed by [14]). In the framework of nerve injury, critical for post-injury phenotypic transitions of SCs from one stage to the next is the repression of genes involved in the antecedent stage, as well as the activation of new genes that will define the succeeding stage [15,16].

In the present study we set out to shed more light into these processes by investigating whether an immune challenge with lipopolysaccharide (LPS) (to mimic inflammation), could trigger dysregulations in the expression profiles of select miRNAs with well-established biological activities in SCs, as well as in the VIP/PACAP neuroprotective system using an in vitro model of cultured rat RT4 SCs. In addition, we performed regression analyses to identify potential correlations between miRNAs and VIP/PACAP system gene expression profiles. Our hypothesis is that following an inflammatory insult, the pattern of selected miRNAs known to be involved in biological functions pertinent to the repair phenotype will be dysregulated in cultured SC lines, whose target genes potentially include members of the endogenous VIP/PACAP system.

## 2. Results

### 2.1. Effects of LPS Treatment on RT4 SCs Viability 

**Figure 1:**
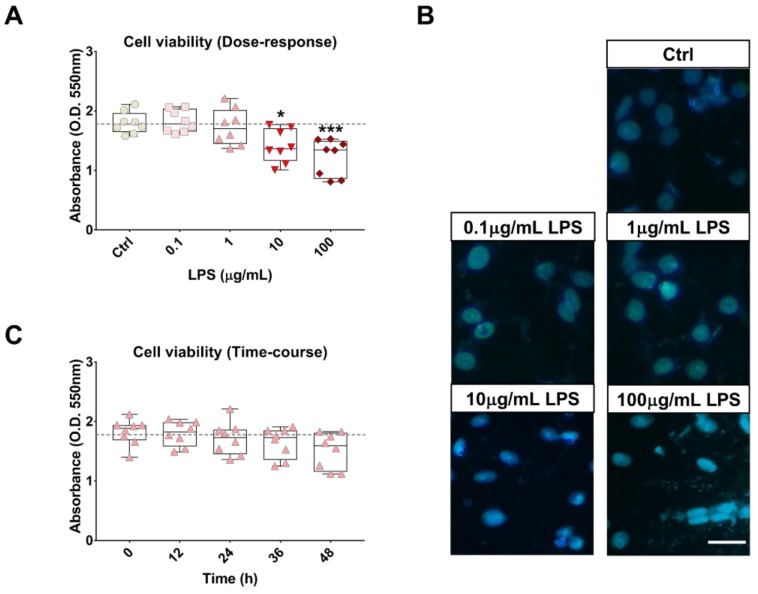
Effects of LPS treatment on RT4 SCs viability. Analysis of cell viability (MTT assay) (**A**) in RT4 SCs grown under normal conditions (Ctrl), or supplemented with increasing concentrations of LPS for 24 h. Values reported represent the mean optical densities (OD) ± S.E.M. from four separate assessments, each run in duplicate using separate cell batches (*n* = 8). * *p* < 0.05 or *** *p* < 0.001 vs. Ctrl, as calculated using One-Way analysis of variance (ANOVA) followed by Dunnett’s *post-hoc* test; (**B**) Hoechst 33258 staining in RT4 SCs treated as in A. Cells were fixed with a solution of methanol/acetic acid (3:1, *v*/*v*) for 30 min, washed three times in PBS and incubated for 15 min at 37 °C with 0.4 μg/mL Hoechst 33,258 dye and analyzed for morphological characteristics of apoptosis under a fluorescence microscope (Axiovert, Zeiss). Original magnification = 63.5×. Scale bar = 20 μm; (**C**) Time-course analyses of cell viability in RT4 SCs exposed to a fixed concentration of LPS (1µg/mL) at different time points (0, 12, 24, 36 and 48 h). Statistical analyses and number of biological replicates as in A.

In order to identify the maximum LPS concentration needed to achieve inflammation in SCs without causing any significant cell death we conducted both dose-response and time-course analyses of cell viability and Hoechst 33258 staining in RT4 SCs treated with LPS. Initially, cells were exposed to increasing concentrations of LPS (0, 0.1, 1, 10 and 100 µg/mL) at a fixed time point of 24 h. As shown in Figure 1 (panel A), LPS concentrations up to 1 µg/mL were devoid of statistically significant effects on cell viability after 24 h (F_4,35_ = 8.608, *p* >0.05, ANOVA followed by Dunnett’s *post-hoc* test). When concentrations were increased by an order of magnitude or two (10 or 100 µg/mL) we observed significant reductions of viability (* *p* = 0.0172 or *** *p* = 0.0003, respectively). To confirm further these findings through morphological observations, we performed Hoechst staining using the same experimental conditions as indicated above. As depicted in Figure 1 (panel B), both 0.1 and 1 µg/mL LPS caused only minor signs of chromatin condensation and no evidence of DNA fragmentation or cell shrinkage, whereas overt signatures of cell death, including cell clustering, were observed in SCs at the highest concentrations tested. Finally, to establish whether the 1 µg/mL LPS concentration was suitable for testing over our planned experimental time window, we run a time-course analysis of cell viability at different time intervals (0, 12, 24, 36 and 48 h) (Figure 1, panel C). One-way ANOVA and *post-hoc* analyses confirmed that no significant reductions of cell viability could be found at any of the time points tested (F_4,35_ = 1.962, *p* > 0.05 at every time points), although proliferation was in part hinder, but still qualifying the 1 µg/mL LPS as the most suitable concentration for the purpose of this study. 

### 2.2. Effects of LPS Treatment on RT4 SCs Inflammatory Profile 

**Figure 2:**
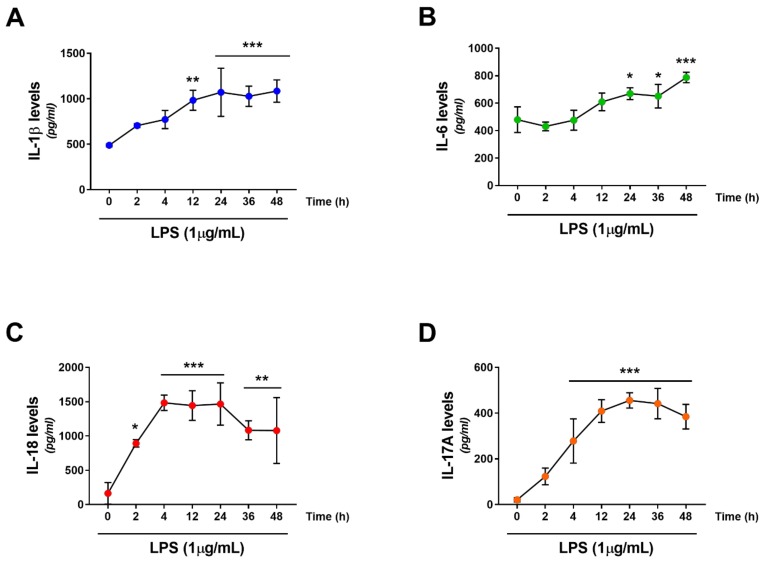

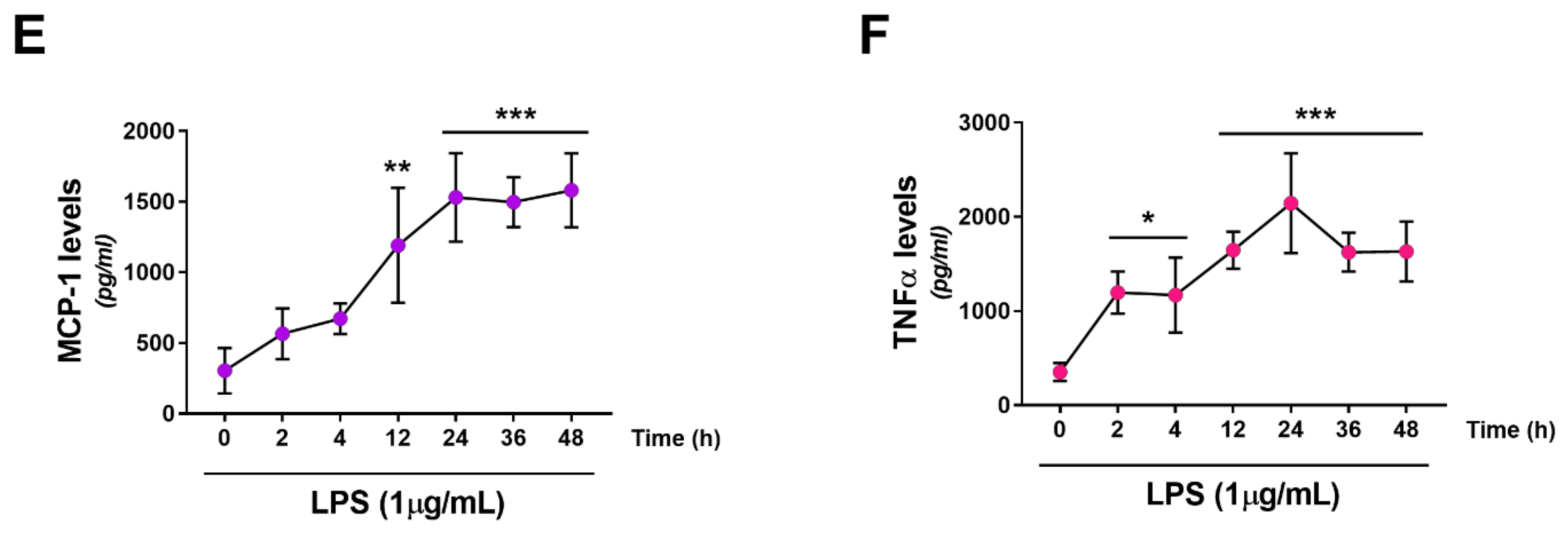
(**A**–**F**) Levels of secreted pro-inflammatory cytokine/chemokines in RT4 SCs treated with LPS at different time points (0, 2, 4, 12, 24, 36 and 48 h) were determined using commercially available Bio-Plex Pro Rat Cytokine I multiplex assay kits (for details refer to Materials and Methods section). Data shown is the result of three independent determinations (*n* = 3), each run in duplicate. * *p* < 0.05, ** *p* < 0.01 or *** *p* < 0.001 vs. time 0, as determined by ANOVA followed by Dunnett’s *post-hoc* test.

Similarly to microglia, SCs express several pattern recognition receptors that allows them to recognize danger signals and respond by secreting cytokines and chemokines, to further attract immune cells to the site of injury [17]. Here, to test the validity of our cellular model in mimicking in vivo SCs responses to inflammatory/danger signals, we opted to test the effects of LPS treatment on the secretion of pro-inflammatory cytokine in our rat RT4 SCs. Customized multiplex cytokine assays were run in RT4 SCs exposed to 1 µg/mL LPS over a range of time points (0, 2, 4, 12, 24, 36 and 48 h, respectively) to assess the levels of 6 different pro-inflammatory cytokines/chemokines: interleukin (IL)-1β, IL-6, IL-18, IL-17A, monocyte chemotactic protein-1 (MCP-1) and tumor necrosis factor-α (TNF-α). Results demonstrated that, albeit at different degrees, LPS treatment remarkably increased the levels of secreted cytokines/chemokines in the culture media (Figure 2, panels A–F). Specifically, a progressive elevation of IL-1β levels was seen over time, with values becoming statistically significant vs. controls after 12 h LPS exposure (F_6, 14_ = 8.93, ** *p* = 0.002; ANOVA followed by Dunnett’s *post-hoc* test) or above (*** *p* < 0.0001). IL-6 required prolonged exposure to LPS (≥24 h) to increase at significant levels (F_6,14_ = 11.68, * *p* = 0.0147 at 24 h, * *p* = 0.028 at 36 h and *** *p* = 0.0003 at 48 h, respectively). IL-18 secretion spiked early following LPS treatment, with significant increases already after 2 h (F_6,14_ = 10.71, * *p* = 0.0137). Cytokine secretion peaked at 4 h (*** *p* = 0.0001), plateaued until 24 h (*** *p* = 0.0001) and then slightly reduced at 36 h (** *p* = 0.0023) and 48 h (** *p* = 0.0024). The levels of IL-17A steadily increased and values were statistically significant as early as after 4 h, peaked at 24 h and then moderately diminished by 48 h (F_6,14_ = 28.01, *** *p* = 0.0001). The pattern of MCP-1 secretion (aka chemokine (C-C motif) ligand 2, CCL-2) was somewhat different. Levels in the media were not much increased up to 4 h post-LPS exposure (F_6,14_ = 13.44, *p* = 0.6333 at 2 h, *p* = 0.3243 at 4 h), but then rapidly augmented at 12 h (** *p* = 0.0032) to reach plateau after 24 h and after (*** *p* = 0.0002). Finally, TNF-α increased at significant levels after 2 h (F_6,14_ = 9.855, * *p* = 0.0234) and maintained steady levels until 4 h (* *p* = 0.0287), to rise again at 12 h (*** *p* = 0.0009) and 24 h (*** *p* = 0.0001) and then slightly attenuate at the following time points (*** *p* = 0.001 both after 36 and 48 h LPS exposure). 

### 2.3. VIP/PACAP System Gene Expression Profile Following LPS Treatment 

**Figure 3:**
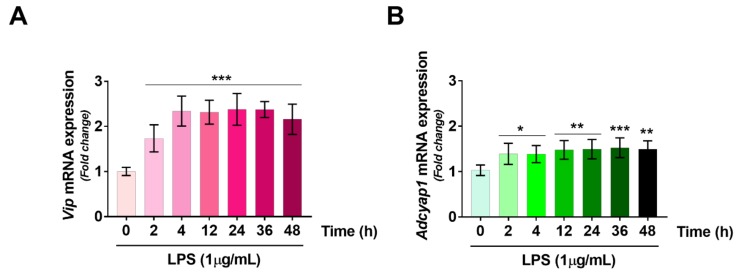

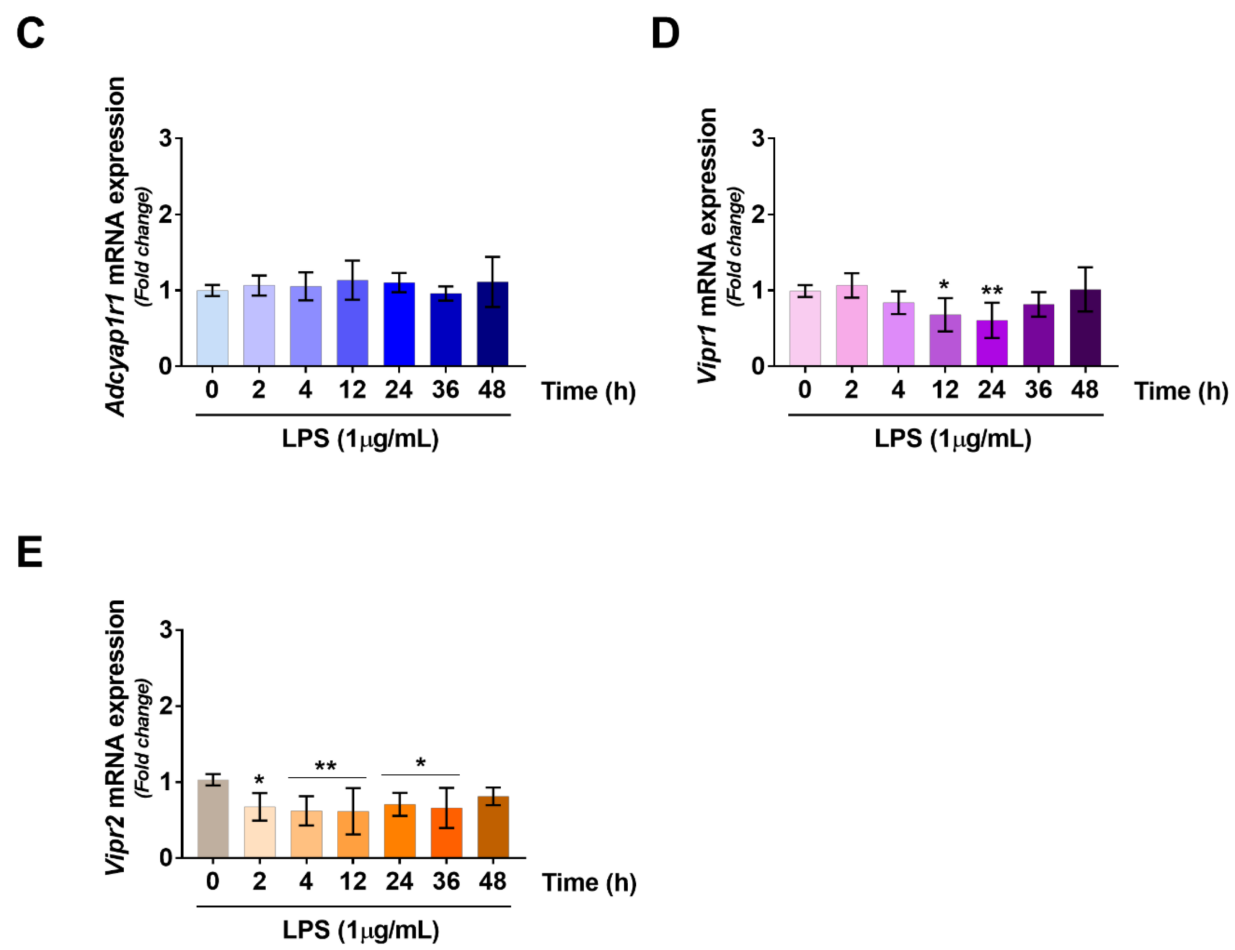
Time-course gene expression profiling of (**A**) *Vip*; (**B**) *Adcyap1*; (**C**) *Adcyap1r1*; (**D**) *Vipr1* and (**E**) *Vipr2* mRNAs in RT4 SCs treated with LPS at the indicated concentration. Relative transcript levels were measured by quantitative real-time PCR analyses. Amplifications were performed using selected primers optimized for qPCR analyses (<150 bp length) recognizing fragments within the CDS of the gene of interest (for details refer to Table 1 below). Results are presented as mean fold changes of time 0 (control) ± SEM.-fold changes of each gene were obtained after normalization to the endogenous ribosomal protein *S18* (reference gene) and then calculated using the comparative ΔΔCt method. Baseline expression levels of control were set to 1. Bar graphs depicted show the mean results obtained from two independent determinations using two separate batches of cells each time (*n* = *4*). * *p* < 0.05, ** *p* < 0.01 or * *p* < 0.001, as determined using ANOVA and Dunnett’s *post-hoc* comparisons.

The VIP/PACAP system is involved in regulating a myriad of biological functions in SCs, including inhibition of apoptosis [6], synthesis of myelin components [4] and production of proteolytic enzymes to provide axonal guidance and digestion of cell debris [5]. Since some of these functions are strongly enhanced following the transition of SCs to a de-differentiated “repair phenotype” [17], we sought to determine the gene expression levels of each VIP/PACAP family component in RT4 SCs following LPS exposure. LPS treatment rapidly induced VIP (*Vip*) gene expression in SCs, by significantly increasing mRNA levels both at 2 h (F_6,35_ = 20.19, *** *p* = 0.0004) and 4 h (*** *p* < 0.0001) to plateau at about 2.3-fold increase thereafter (*** *p* < 0.0001) (Figure 3, panel A). Despite significant, PACAP (A*dcyap1*) gene levels exhibited a more blunted increase in response to LPS, with an average overall increase of ~1.4–1.5-fold when compared with controls over the entire time-window tested (F_6,35_ = 4.507, * *p* = 0.0163 at 2 h, * *p* = 0.0189 at 4 h, ** *p* = 0.0021 at 12 h, ** *p* = 0.0014 at 24 h, *** *p* = 0.0007 at 36 h and ** *p* = 0.0015 at 48 h) (Figure 3, panel B). Expression of the PACAP-preferring PAC1 receptor (*Adcyap1r1*) gene was unaffected by treatment at any of the time points assessed (F_6,35_ = 0.6618, *p* = 0.6618) (Figure 3, panel C). Conversely, both VPAC1 (*Vipr1*) and VPAC2 (*Vipr2*) mRNAs were significantly downregulated by the immune challenge. More specifically, *Vipr1* transcripts were significantly lower than controls after 12 h (0.68-fold, F_6,35_ = 4.801, * *p* = 0.0414) and 24 h (0.61-fold, ** *p* = 0.0081) to slowly recover to control levels after 36 and 48 h (0.82 and 1.01-fold, *p* = 0.4453 and 0.9997, respectively) (Figure 3, panel D). *Vipr2* reduction happened earlier when compared with *Vipr1*, with significantly reduced gene expression as early as after 2 h LPS exposure (0.67-fold, F_6,35_ = 3.34, * *p* = 0.0178) and lasting until 36 h (0.62-fold, ** *p* = 0.0054 at 4 h, 0.61-fold, ** *p* = 0.0046 at 12 h, 0.71-fold, * *p* = 0.0364 at 24 h and 0.66-fold, * *p* = 0.0127 at 36 h, respectively) to finally return almost to baseline levels by 48 h (0.81-fold, *p* = 0.2493) (Figure 3, panel E). 

### 2.4. VIP/PACAP System Protein Expression Profile Following LPS Treatment 

**Figure 4:**
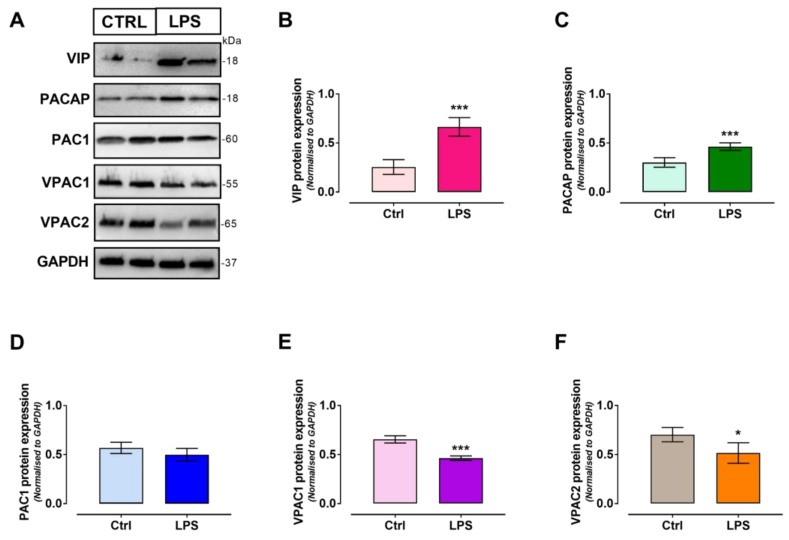
(**A**) Representative Western blots showing the effects of 24 h exposure to 1 µg/mL LPS in RT4 SCs. Semi-quantitative analyses of bands’ intensities show significant increases both in (**B**) VIP and (**C**) PACAP protein expression levels, but not in (**D**) PAC1 receptor, which was unaffected by treatment. Conversely, both (**E**) VPAC1 and (**F**) VPAC2 receptor levels were diminished. Data are the mean ± SEM of two experiments, each using 2 separate batches of cells per group (*n* = 4). * *p* < 0.05 or *** *p* < 0.0001 vs. Ctrl; Unpaired Student *t*-test.

To investigate whether the expression of each of the components of the VIP/PACAP system was perturbed in response to LPS exposure, Western blot analyses were carried out in cells after 24 h treatment. Results are depicted in Figure 4 below. As demonstrated by semi-quantitative analyses of blots intensities, treatment with LPS caused a significant increase in the expression of both VIP (t_6_ = 6.77, *** *p* = 0.0005, as determined using the unpaired Student *t*-test) and to lesser extent, of PACAP protein levels compared with untreated controls in SCs (t_6_ = 5.207, *** *p* = 0.002) (Figure 4, panels A–C). Similarly to mRNA data, PAC1 expression was unaffected by treatment (t_6_ = 1.627, *p* = 0.1548) whilst VPAC1 and VPAC2 were significantly reduced (VPAC1→ t_6_ = 8.794, *** *p* = 0.0001; VPAC2→t_6_ = 2.936, * *p* = 0.0261, respectively) (Figure 4, panels A, D–F). 

### 2.5. Dynamics of miRNAs Expression in RT4 SCs Exposed to LPS Treatment 

**Figure 5:**
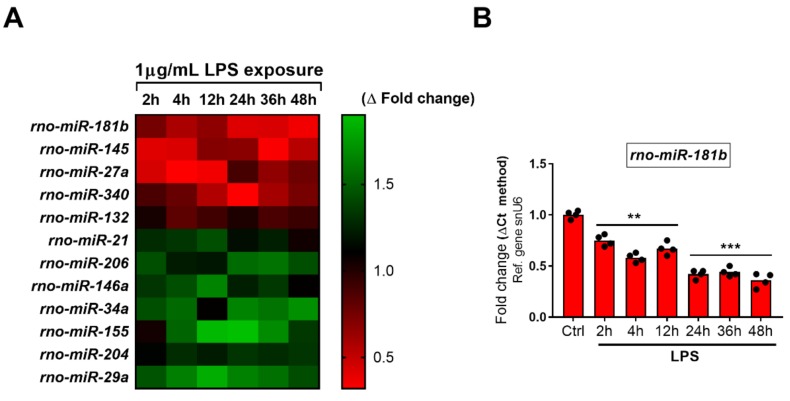

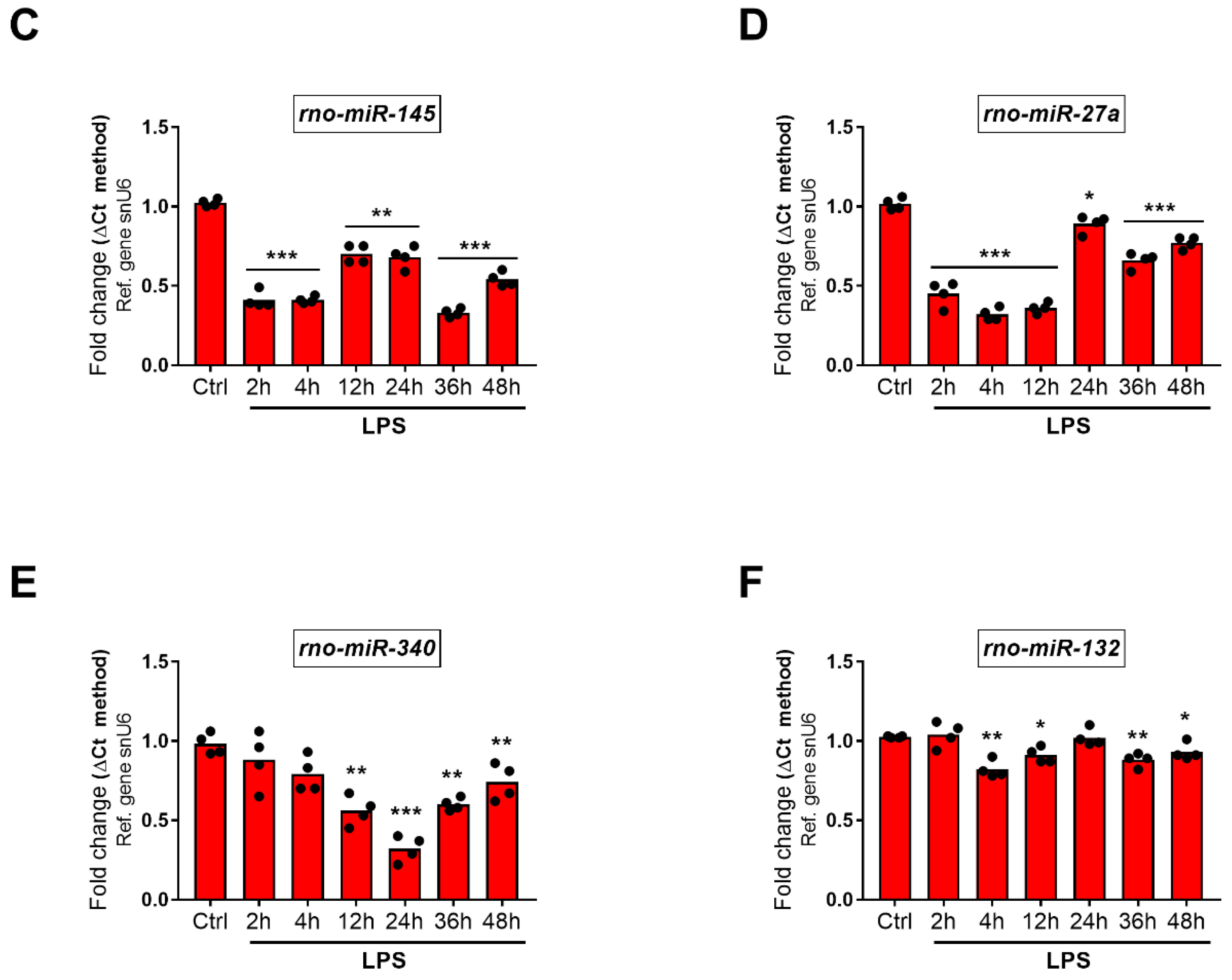
Time-course analyses of downregulated miRNAs following LPS exposure. (**A**) Heatmap showing the overall pattern of expression of the dysregulated miRNAs over time. Each bar graph depicts the expression profile of (**B**) *rno-miR-181b*; (**C**) *rno-miR-145*; (**D**) *rno-miR-27a*; (**E**) *rno-miR-340* and (**F**) *rno-miR-132* after treatment with 1 µg/mL LPS at different time points (0, 2, 4, 12, 24, 36 and 48 h). Ctrl = time 0. Data shown are the mean fold change ± SEM, obtained from two independent experiments, each performed using separate biological replicates (*n* = 4). * *p* < 0.05, ** *p* < 0.01 or *** *p* < 0.001 vs. Ctrl; repeated measures ANOVA followed by Fisher’s LSD test.

**Figure 6:**
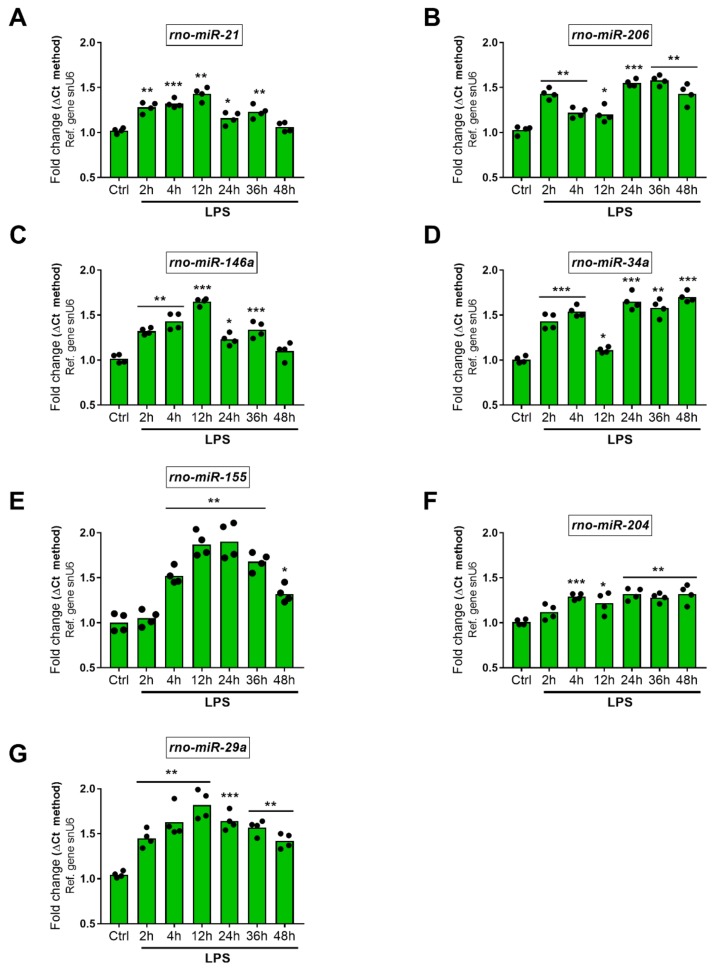
Time-course analyses of upregulated miRNAs following LPS exposure. Each bar graph depicts the expression profile of (**A**) *rno-miR-21*; (**B**) *rno-miR-206*; (**C**) *rno-miR-146a*; (**D**) *rno-miR-34a*; (**E**) *rno-miR-155*; (**F**) *rno-miR-204* and (**G**) *rno-miR-29a* after treatment with 1 µg/mL LPS at different time points (0, 2, 4, 12, 24, 36 and 48 h). Ctrl = time 0. Data shown are the mean fold change ± SEM, obtained from two independent experiments, each performed using separate biological replicates (*n* = 4). * *p* < 0.05, ** *p* < 0.01 or *** *p* < 0.001 vs. Ctrl; repeated measures ANOVA followed by Fisher’s LSD test.

With the idea of investigating the expression profile of select miRNAs previously shown to regulate certain functions of SCs pertinent to the post-injury transition to the “repair phenotype” in our cell model, we first conducted a thorough literature research in the attempt to identify the most relevant candidates. As shown in Table 2, we identified 18 putative miRNA candidates. Downstream time-course analyses of gene expression by RT-qPCR demonstrated that only 12 of the identified miRNAs were significantly dysregulated by LPS treatment in our cell system (Figure 5 and Figure 6 below), whereas six miRNAs were not significantly affected.

The heatmap depicted in Figure 5 (panel A) illustrates the pattern of expression of the dysregulated miRNAs, characterized by the presence of 5 significantly downregulated (i.e., *rno-miR-181b, -145, -27a, -340* and *-132*) and 7 significantly upregulated miRNAs (i.e., *rno-miR-21, -206, -146a, -34a, -155, -204* and *-29a*). 

#### 2.5.1. Downregulated miRNAs

Repeated measures ANOVA followed by Fisher’s LSD test demonstrated that *rno-miR-181b* was significantly diminished after 2 h LPS (0.75-fold vs. Ctrl, F_6,27_ = 73.95, ** *p* = 0.0052) and further reduced till the 48 h time point (0.58-fold at 4 h, ** *p* = 0.0012; 0.67-fold at 12 h, ** *p* = 0.0069; 0.42-fold at 24 h, *** *p* = 0.0005, 0.44-fold at 36 h, *** *p* = 0.0008 and 0.36-fold at 48 h, *** *p* = 0.0004, respectively). *Rno-miR-145* levels were robustly reduced after 2 and 4 h (F_6,27_ = 96.44, 0.41-fold both at 2 h, *** *p* = 0.0004 and 4 h, *** *p* < 0.0001), slightly increased at 12 and 24 h (0.7-fold, ** *p* = 0.0021 and 0.68-fold, ** *p* = 0.002) to drop down again at the later times tested (0.33-fold at 36 h, *** *p* < 0.0001 and 0.54-fold at 48 h, *** *p* = 0.0003). The expression of *rno-miR-27a* reduced to less than 0.5-fold of control after 2, 4 and 12 h treatment (F_6,27_ = 140.8, *** *p* = 0.0006 at 2 h and *** *p* < 0.0001 at 4 h and 12 h, respectively). After 24 h, the decrease was much less evident with respect to controls, though still significant (0.89-fold, * *p* = 0.0194), but decreased further at 36 h (0.66-fold, *** *p* = 0.0009) and 48 h (0.77-fold, *** *p* < 0.0001). *Rno-miR-340* pattern of expression was characterized by a slow and steady decrease, which became statistically significant only after 12 h (0.56-fold, F_6,27_ = 17.83, ** *p* = 0.0071), reached the minimum at 24 h (0.32-fold, *** *p* = 0.0009) to slightly augment thereafter (0.6-fold at 36 h, ** *p* = 0.0039 and 0.74-fold at 48 h, ** *p* = 0.0054). Regarding *rno-miR-132*, the time-course of gene expression showed moderate fluctuations, with an initial significant downregulation at 4 and 12 h (F_6,27_ = 9.28, 0.82-fold, ** *p* = 0.0042 and 0.91-fold, * *p* = 0.0243) followed by a return to baseline at 24 h (1.02-fold, *p* = 0.8741) and again a modest reduction at 36 and 48 h (0.88-fold, ** *p* = 0.0049 and 0.93-fold, * *p* = 0.0353) (Figure 5, panels B–F). 

#### 2.5.2. Upregulated miRNAs

*Rno-miR-21* was upregulated at significant levels after 2 and 4 h LPS treatment (F_6,27_ = 23.76, 1.28-fold, ** *p* = 0.0016 and 1.32-fold, *** *p* = 0.0009). Expression peaked at 12 h (1.43-fold increase, ** *p* = 0.0025) but diminished at 24 and 36 h (1.16-fold, * *p* = 0.0364 and 1.23-fold, ** *p* = 0.0061) to finally return to baseline levels at 48 h (1.06-fold, *p* = 0.2235). Expression of *rno-miR-206* increased about 1.5-fold at 2 h (F_6,27_ = 37.66, ** *p* = 0.0017), lowered to about 1.2-fold at 4 and 12 h (** *p* = 0.0062 and * *p* = 0.0184) and raised again at 24, 36 and 48 h (1.55-fold at 24 h *** *p* = 0.0002, 1.58-fold at 36 h ** *p* = 0.0014 and 1.43-fold at 48 h ** *p* = 0.0019). *Rno-miR-146a* increased significantly after 2, 4 h (F_6,27_ = 41.61, 1.32-fold, ** *p* = 0.0014 and 1.43-fold, ** *p* = 0.0044) and reached its peak of expression by 12 h (1.65-fold, *** *p* = 0.0003), followed by a downwards slope at 24, 36 and 48 h (1.23-fold, * *p* = 0.012; 1.34-fold, ** *p* = 0.0007 and 1.1-fold, *p* = 0.1773). LPS induction of *Rno-miR-34a* was observed both at 2 and 4 h post-LPS exposure (F_6,27_ = 62.62, 1.43-fold, *** *p* = 0.0006 and 1.54-fold, *** *p* = 0.0002, respectively), decreased but was still significantly upregulated after 12 h (1.11-fold * *p* = 0.0418) to then further increase at later time points (1.65-fold at 24 h, *** *p* = 0.0005; 1.58-fold at 36 h, ** *p* = 0.0014; 1.7-fold at 48 h, *** *p* = 0.0005). The expression profile of *rno-miR-155* followed an inverted U-shape, with transcripts significantly upregulated up to 24 h (F_6,27_ = 35.18, 1.05-fold at 2 h, *p* = 0.5803; 1.52-fold at 4 h, ** *p* = 0.008; 1.87-fold at 12 h, ** *p* = 0.0027 and 1.9-fold at 24 h, ** *p* = 0.0084) to gradually attenuate at 36 and 48 h (1.68-fold Vs. control, ** *p* = 0.0018 and 1.32-fold, * *p* = 0.026, respectively). With regards to *rno-miR-204*, the overall increase was moderate but statistically significant, with a peak induction of 1.32-fold observed both after 24 and 48 h exposure to LPS (F_6,27_ = 9.173, ** *p* = 0.0041 and 0.0088, respectively). A gradient increase in *rno-miR-29a* was observed in SCs exposed to LPS. Transcripts were increased 1.45-fold at 2 h (F_6,27_ = 20.72, ** *p* = 0.0012), 1.63-fold at 4 h (** *p* = 0.0041), 1.82-fold at 12 h (** *p* = 0.0019), followed by a more blunted upregulation from 24 h onwards (1.64-fold at 24 h, *** *p* = 0.001; 1.57-fold at 36 h, ** *p* = 0.0011 and 1.42-fold at 48 h, ** *p* = 0.0063) (Figure 6, panels A–G).

### 2.6. Correlations between Dysregulated miRNAs and VIP/PACAP System mRNAs in LPS Exposed RT4 SCs

**Figure 7:**
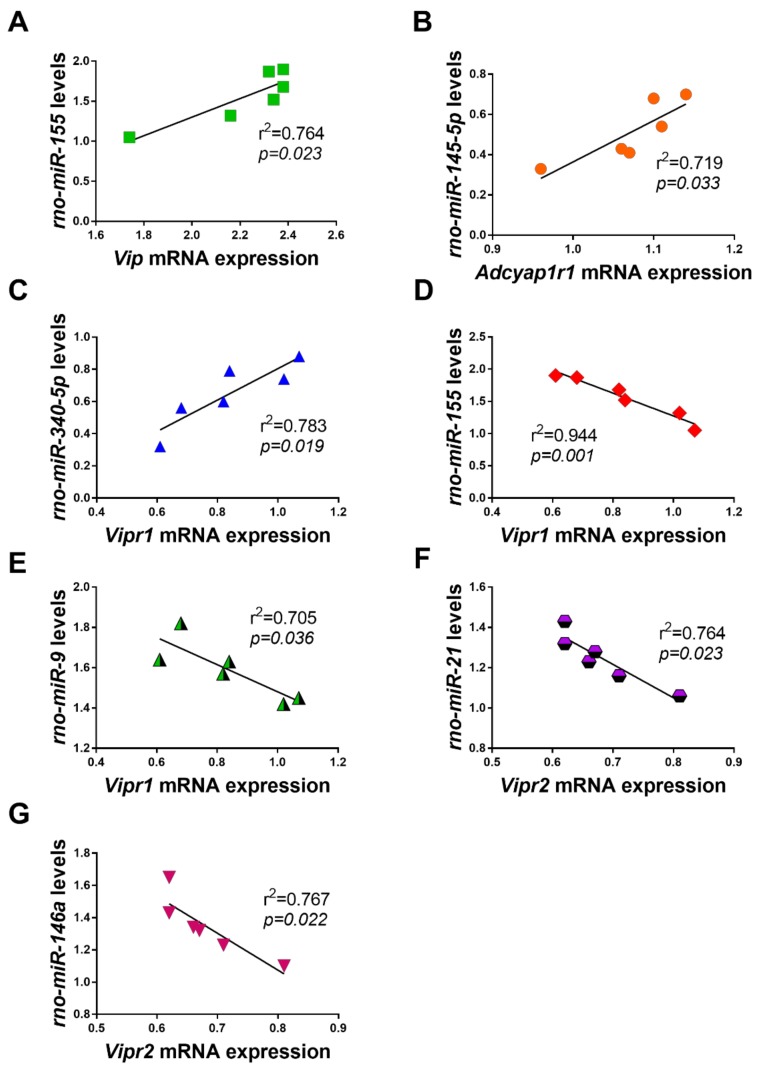
Scatterplots and regression lines showing the relationship between dysregulated miRNAs and the expression profile of VIP/PACAP system components following exposure to LPS. Only significant correlations are shown. Positive correlations were found between (**A**) *rno-miR-155* and *Vip*; (**B**) *rno-miR-145-5p* and *Adcyap1r1* and (**C**) *rno-miR-340-5p* and *Vipr1* mRNAs. Inverse relationships were found between (**D**) *rno-miR-155* and *Vipr1*; (**E**) *rno-miR-29a* and *Vipr1*; (**F**) *rno-miR-21* and *Vipr2* and (**G**) *rno-miR-146a* and *Vipr2*. Coefficients of determination (*r*^2^) and related p values are shown in each panel.

In the effort to define if the pattern of changes of the dysregulated miRNAs correlated with the changes occurred in the VIP/PACAP system in our cell model, we sought to conduct linear regression analyses. Only significant correlations are shown (*p* < 0.05). We found three positive (Figure 7, panels A–C) and four inverse correlations (Figure 7, panels D–G) with the VIP/PACAP family components. None of the investigated miRNAs correlated with *Adcyap1* (PACAP gene) transcripts in SCs treated with LPS the expression. On the other hand, *rno-miR-155* showed a significant positive correlation with *Vip* mRNAs (*r*^2^ = 0.764, *p* = 0.023) and so did *rno-miR-145* with the PACAP-preferring receptor *Adcyap1r1* mRNAs (*r*^2^ = 0.719, *p* = 0.033). Noteworthy, among the dysregulated miRNAs, 1 significant positive and 2 inverse correlations were found with *Vipr1* transcripts: *rno-miR-340* (*r*^2^ = 0.783, *p* = 0.019), *rno-miR-155* (*r*^2^ = 0.944, *p* = 0.001) and *rno-miR-29a* (*r*^2^ = 0.705, *p* = 0.036). Finally, two inverse relationships were computed for *Vipr2* gene: with *rno-miR-21* (*r*^2^ = 0.764, *p* = 0.023) and *rno-miR-146a* (*r*^2^ = 0.767, *p* = 0.022). 

## 3. Discussion

In this study, we have unveiled a subset of miRNAs whose expression levels are dysregulated in RT4 SCs in vitro when exposed to an immune challenge with LPS. We performed these investigations in the attempt to gain mechanistic insights that could aid in modelling the cell behavior of SCs in vivo when exposed to the local inflammatory microenvironment found following nerve injury. The panel of miRNAs we analyzed was selected on the basis of prior evidence documenting their specific involvement in regulating certain biological functions relevant to repair SCs (please refer to Table 2 above for details), a de-differentiated phenotype that SCs acquire to aid in the nerve regeneration process following damage. In addition, we conducted regression analyses and identified significant correlations among some of the perturbed miRNAs profiles and the expression pattern of components of the neuroprotective VIP/PACAP system. To the best of our knowledge, this is the first study to: (1) detect specific changes in the expression pattern of VIP/PACAP system following LPS exposure in rat RT4 SCs and (2) identify a subset of dysregulated miRNAs in RT4 SCs whose expression pattern correlates with changes to the VIP/PACAP system. 

The current investigations were conceived based on previous findings demonstrating that the VIP/PACAP system is involved in regulating multiple biological actions in RT4 SCs. Some of these functions included promoting the shift to a proliferative state through the inhibition of programmed cell death [6] or the induction of gene expression and activity of a class of proteolytic enzymes involved in the clearance of cell debris along the nerve sheath conduit [5], a condition necessary for axonal regeneration. We reasoned that such a variety of biological functions in SCs could not be simply the result of the ligand-receptor operated activation of transduction systems, but likely involved a more subtle and complex machinery able to fine-tune and coordinate the expression of a whole set of genes. MiRNAs, which appeared on the scene just less than two decades ago [12], are tiny non-coding RNAs that control several pathways through the suppression of hundreds of genes and, in turn, are reciprocally controlled by various regulatory networks [39]. As evidence suggests, these post-transcriptional repressors of gene expression appeared the most suitable targets to investigate in our cell model.

Initially, our approach was to establish the suitability of our cell system as an effective in vitro model that could at least in part mimic the biological response of SCs to an “immune challenge”. Hence, we performed a series of experiments aimed at assessing which was the optimal concentration of LPS to use as well as the correct time window of exposure (Figure 1). Secondly, we assessed whether LPS triggered the release of pro-inflammatory cytokines/chemokines by RT4 SCs (Figure 2). Surprisingly, we found that RT4 SCs are resilient to the detrimental effects of LPS, and signs of apoptosis were apparent only at reasonably high concentrations (above 10 µg/mL) after 24 h. We could not ascertain the exact mechanisms for such increased cell resistance, but based on prior observations we assumed that the endogenous upregulation of the protective peptides VIP and PACAP might have had a role (Figure 3 and Figure 4), with a similar process to that observed in primary SCs exposed to oxidative insult [40]. Regarding the inflammatory response, LPS-stimulated RT4 SCs secreted higher levels of several pro-inflammatory cytokines/chemokines, albeit each reaching their individual peak of secretion at different times. Noteworthy, of all the tested cytokines/chemokines, which peaked at around 12–24 h post-LPS, IL-18 reached its peak as early as after 4 h. This finding is in agreement with studies supporting the important role of this cytokine in priming neutrophil recruitment at the earliest stages of inflammation [41] to promote adaptive immune cell recruitment thereafter [42]. Moreover, the overall increase in cytokine levels is consistent with the idea of an active involvement of SCs in promoting the recruitment of macrophages and resident glia at the injury site [17,43].

### 3.1. Dysregulated Expression of the VIP/PACAP System upon LPS Exposure 

Both VIP and PACAP are two well-established naturally occurring peptides endowed with neuro- and glioprotective properties [44]. Due to their protective function, endogenous levels of VIP and PACAP are usually upregulated in response to changes in microenvironment that would otherwise damage or kill cells, most likely as a self-preservation mechanism. However, the peptides are also pivotal in a variety of other important biological functions, some of which seem to be cell/tissue type specific [45,46,47]. In cultured RT4 SCs, both peptides inhibit apoptosis [6], trigger fibrinolytic activity [5], stimulate the production of other trophic molecules [40] and ultimately promote myelinogenesis [4]. Here we found that upon LPS stimulation, VIP and PACAP transcripts and proteins were significantly increased, whilst unexpectedly, the expression of VPAC1 and VPAC2 receptors was diminished. Based on findings reporting that the actions of VIP/PACAP are through autocrine/paracrine signaling loops [48,49], we reasoned that the inverse relationship between peptides and receptors could reflect a regulatory mechanism where inflammation triggers receptors internalization and arrests the release of peptides. This would occur in the effort to promote the intracellular accumulation of both VIP and PACAP at the benefit of other cellular functions, whilst preserving bioenergy consumption. Alternatively, it is possible that the inversely regulated expression of VIP/PACAP peptides and VPAC receptor subtypes is needed to dampen some of the functions normally elicited in SCs, such as the pro-myelinating properties of the peptides, where both peptides and receptors need to be upregulated to induce the expression of myelin-markers [4]. However, further investigations are warranted to verify these hypotheses.

### 3.2. Dysregulated miRNAs and Their Potential Relationship with the VIP/PACAP System 

SCs surrounding damaged axons have the ability to undergo a de-differentiation program that is driven by a transcriptional program that is critical for nerve regeneration. In part, this program is thought to be initiated during the earliest phase of nerve injury, when overt signs of inflammation are observed at the injury site. Evidence suggests that miRNAs might play a central role in controlling cellular reprogramming of SCs [50]. According to an emerging theory, the multifaceted process of de-differentiation in SCs is controlled by the interplay of cell-intrinsic programs and cell-extrinsic signals. Immediately after injury, degenerating axons release signaling molecules to trigger SC de-differentiation, presumably through the activation of cell-intrinsic transcriptional programs coordinated by miRNAs. Some of these programs are known to be partaken by transcription factors such as c-jun [51], DNA cofactors such as the proliferating cell nuclear antigen (PCNA) [52] and different dedifferentiating receptors, including platelet-derived growth factor (PDGF) B [53]. Extrinsic pro-inflammatory signals from the microenvironment would then superimpose on these programs to adapt SC function to the specific repair requirements of the surrounding tissue [10]. In line with this theory, this study revealed that at least 5 of the selected miRNAs were significantly downregulated and 7 were upregulated using our experimental model.

As previously validated by other research groups (please refer to Table 2 for details) the functions of the dysregulated miRNAs ranged from the control of cell proliferation (*miR-27a*), cell migration (*miR-132* and *miR-29a*), myelinogenesis (*miR-29a*), apoptosis (*miR-204*), response to inflammation (*miR-155*) and clearance of debris (*miR-340*), to the control of de-differentiation genes (*miR-34a*, *miR-145* and *miR-181b*), all collectively involved in the phenotypic regression of SCs to a proliferative state. Interestingly, some of the miRNAs were affected by LPS treatment in a manner consistent with that previously reported to attain the expected biological shifts. For instance, miR-340, which directly targets the 3′ untranslated region (UTR) of the proteolytic enzyme tissue plasminogen activator gene (*tPA*) [38], was significantly downregulated in RT4 SCs exposed to LPS; suggesting that upon inflammatory stimuli SCs may increase their proteolytic activity to promote debris clearance and extracellular matrix remodeling by de-repressing *tPA* gene expression. Another downregulated miRNA in this study, i.e., *miR-181b*, has been previously shown to repress the release of pro-inflammatory cytokines in astrocytes [32]. Despite the difference in cell type, it is likely that the attenuation of *miR-181* may exert a comparable function in SCs to boost cytokine release.

Of the 7 aberrantly upregulated miRNAs, 2 were more predominantly increased, i.e., *miR-155* and *miR-29a* (Figure 6, panels E, G). *MiR-155* increase has been implicated in the pathogenesis of various inflammatory disorders [31], including the triggering of autoimmune inflammation by T cells [54] and potentiation of LPS-induced release of pro-inflammatory cytokines by monocytes in cells exposed to hypothermia [55]. In addition, this miRNA seems to be regulated following activation of toll-like receptors [31], a family of pattern recognition receptors that also recognize and are activated by LPS. Collectively, these findings may imply that the increased *miR-155* might be partly responsible for the release of pro-inflammatory mediators in LPS-exposed RT4 SCs. The other highly upregulated miRNA, *miR-29a*, is involved in the suppression of peripheral myelin protein 22 (PMP22) gene in SCs [20]. PMP22 is an essential component of the myelin sheath and is required for myelinogenesis. *MiR-29a* driven gene repression of PMP22 could halt SC pro-myelinating function during inflammation, thereby promoting the transition to a non-myelinating phenotype. 

Taken together, the observed patterns of dysregulations in the investigated miRNAs may explain, at least in part, some of the biological changes SCs might undergo following exposure to an inflammatory trigger. Due to the number of gene targets regulated by these non-coding RNAs, it is of no surprise that the coordinated activity of a few miRNAs could potentially generate important changes in cell functioning and biology. With this in mind, we decided to take a step further and analyze our data in the attempt to find, if any, a correlation with the temporal gene expression profile of genes pertaining to the VIP/PACAP system. Unfortunately, we could not find any significant relationship between our miRNAs and one of the dysregulated genes, the PACAP gene. However, we identified 3 positive correlations; one between *miR-155* and *Vip* transcripts, the second between *miR-145* and PAC1 receptor gene (*Adcyap1r1*) and the third one between *miR-340* and VPAC1 gene (*Vipr1*) (Figure 7, panels A–C). These relationships were found to be quiet unusual in view of the suppressive nature of miRNAs on transcripts, which usually results in inverse correlations. However, it is not always the case. As elegantly described by Dugas and Notterpek [56], some miRNAs may function as “guardians of the transcriptome” by preventing inappropriately expressed mRNAs from being translated into functional proteins that could otherwise affect the health of the cell. In our setting, it is possible that uncontrolled induction of VIP, PAC1 and VPAC1 receptor genes by LPS would have impeded the appropriate cell response to inflammation. *MiR-155*, *-145* and *-340*, by targeting these “inappropriate genes” may have prevented or limited excessive translation. Although not confirmed by direct gene targeting testing, this perspective appears fascinating. On the other hand, two couples of miRNAs per gene (i.e., *miR-155/-29a* vs. *Vipr1* and *miR-21/-146a* vs. *Vipr2* genes, respectively) exhibited the canonical inverse relationships with their putative targets, hence providing a plausible explanation for the robust reduction of protein levels we reported. 

In conclusion, the present study demonstrated that an inflammatory insult, mimicked by LPS, causes significant dysregulations of two biologically relevant and potentially interrelated systems in SCs. However, it should be noted that the significance of these findings in the broader context of nerve injury should be taken with care in view of the fact that the studies have been carried out in RT4 SCs and not in primary cell lines. With this in mind, further studies are required to investigate whether primary cultures of SCs isolated from injured peripheral nerves would exhibit a similar pattern of dysregulations as that observed in this study. In addition, the involvement of the VIP/PACAP system in the reprogramming of SCs still needs to be comprehensively explored. Additional studies aimed at investigating for potential correlations between the identified miRNAs and better validated targets such as c-jun, PCNA, PDGF receptors are warranted in the future. Nonetheless, these findings may aid in the identification of post-transcriptional mechanisms whose dysregulations could be critical in enabling or determining SCs shift from myelinating to “repair cells” following damage, which could become targets for intervention to aid in promoting peripheral nerve recovery following trauma. 

## 4. Materials and Methods

### 4.1. RT4 Schwann Cell Line 

The present study was performed using the rat Schwann cell line RT4-D6P2T (ATCC number CRL-2768) obtained from the American Type Culture Collection (Rockville, MD, USA). Cells were cultured in Dulbecco’s modified Eagle’s medium (DMEM) and supplemented with 10% of heat-inactivated fetal bovine serum (FBS), 100 U/mL penicillin, and 100 μg/mL streptomycin (Lonza, Milan, Italy). Cells were incubated at 37 °C in a humidified atmosphere with 5% CO_2_. Cells were grown to reach about 80–85% confluence in media containing 10% fetal bovine serum (FBS) before treatment with lipopolysaccharides (LPS) from Escherichia coli O111:B4 (cat no# L4391, Sigma Aldrich, St. Louis, MO, USA) at the concentrations and time regimens described in the related subsections. LPS was diluted in water from the stock solution and stored at −80 °C until needed.

### 4.2. Cell Viability Assay (3-(4,5-Dimethylthiazol-2-yl)-2,5-Diphenyltetrazolium Bromide Assay) 

To assess cell viability, we used the cell proliferation kit I (MTT, Roche Applied Science, Monza, Italy) according to previously reported protocols [57]. RT4 cells were treated with a range of concentrations (0, 0.1, 1, 10 and 100 µg/mL) of LPS (Sigma Aldrich) for 24 h or at the fixed concentration of 1µg/mL for time-course studies. Briefly, cells were seeded into flat bottom 96-well plates at a concentration of 1 × 10^5^ cells/well. DMEM containing 0.5 mg/mL 3-(4,5-dimethylthiazol-2-yl)-2,5-diphenyltetrazolium bromide (MTT) (Sigma Aldrich) was added in each well. Following incubation for 2 h at 37 °C, medium was removed, and 100 μL of dimethyl sulphoxide (DMSO) was added. Formazan obtained by the cleavage of the yellow tetrazolium salt MTT was measured using a spectrophotometer by absorbance change at 550–600 nm using a microplate reader (BioRad, Milan, Italy). 

### 4.3. Hoechst 33258 Nuclear Staining

To appraise the typical morphological features of apoptotic degeneration by fluorescence microscopy we stained RT4 cells with the nuclear dye Hoechst 33258 as previously described [40]. Cells were fixed with a solution containing methanol/acetic acid (3:1 *v*/*v*) for 30 min, then washed three times in PBS and incubated for 15 min at 37 °C with 0.4 μg/mL Hoechst 33258 dye (Bis-benzimide). After being rinsed in milliQ water, cells were visualized for determination of nuclear chromatin morphology using the Axiovert 40 fluorescence microscope (Carl Zeiss Inc., Jena, Germany). Apoptotic/dead cells were recognized on the basis of nuclear shrinkage and/or chromatin fragmentation. Each condition was replicated three times in each experiment. Apoptotic and normal cells were recognised by analyzing three different fields *per* dish following a fixed pattern. 

### 4.4. Multiplex Cytokine Assays

Pre-blended customized multiplex cytokine assays (Bio-Plex Pro Rat Cytokine I 6plx XPL, Bio-Rad Laboratories, Milan, Italy) were used, according to the manufacturer’s instructions, to determine the concentration of IL-1β, IL-6, IL-18, IL-17A, MCP-1 and TNFα in conditioned media from control and LPS-treated Schwann cells at different time points (0, 2, 4, 12, 24, 36 and 48 h). Samples were mixed 1:2 in diluent and stored at 4 °C until used. To start the assay, 50 μL of vortexed magnetic microbeads were added to each well. Beads were then washed twice with Bio-Plex Pro wash buffer using a magnetic plate washer (Tecan HydroFlex, Crailsheim, Germany). 50 μL of standards and samples were added to the wells. Plates were kept in the dark before being incubated at room temperature for 1 h on a plate mixer. Plates were washed thrice and 25 μL of detection antibodies were added and allowed to incubate for 30 min. Plates were washed 3 further times and 60 μL of streptavidin-phycoerythrin reporter (SA-PE) was added to each well, followed by 10 min incubation. Next, plates were washed three times before adding 125 μL assay buffer to each well. Plates were then sealed and stored at 4 °C until acquisition. Data were collected using a Bio-Plex 100 suspension array system (Bio-Rad Laboratories, Milan, Italy). Plates were shaked at 1000 rpm for 30 s prior to reading. Standard curves were optimised and sample cytokine concentrations determined using Bio-Plex Manager software (Bio-Rad Laboratories). 

### 4.5. RNA Isolation, cDNA Synthesis and Gene Expression Analyses by Quantitative Real-Time PCR 

Total RNAs from RT4-D6P2T cells exposed to different treatments were isolated using 1 mL TRIzol reagent (Invitrogen, Monza, Italy) and 0.2 mL chloroform and precipitated with 0.5 mL isopropanol. Pellets were washed with 75% ethanol and air dried. MiRNAs were extracted from cells pellets using the Qiagen miRNeasy mini kit (Qiagen, Hilden, Germany) and finally eluted in 40 µL of elution buffer, according to manufacturer’s instructions. Single stranded cDNAs were synthesized by incubating total RNA (2 μg) with SuperScript III RNase H-reverse transcriptase (200 U/μL) (Invitrogen); Oligo-(dT)_20_ primer (100 nM) (Invitrogen); 1 mM dNTP mix (Invitrogen), dithiothreitol (DTT, 0.1 M), recombinant RNase-inhibitor (40 U/μL) at 42 °C for 1 h in a final volume of 20 μL. Reaction was terminated by incubation of samples at 70 °C for 10 min.Aliquots of cDNA (100 ng) from each sample and external standards (purified amplicons, ranging from 10^2^ to 10^8^ copies) were amplified in parallel reactions, according to previously detailed protocols [58]. Briefly, primer pairs were designed and optimised to specifically target *Vip, adcyap1, adcyap1r1, Vipr1, Vipr2* the *S18* ribosomal subunit (reference gene) coding regions mRNAs, using the sequences detailed in Table 1. Each PCR reaction contained 0.5 μM primers, 1.6 mM MgCl^2+^, 1× (Roche Diagnostic, Monza, Italy). Amplifications were performed using the Light Cycler 480 instrument (Roche Diagnostic) using the following program setting: (1) cDNA denaturation (1 cycle: 95 °C for 2 min); (2) quantification (45 cycles: 95 °C for 10 s, 60 °C for 30 s, 72 °C for 7 s); (3) melting curve analysis (1 cycle: 95 °C for 0 s, 65 °C for 15 s, 95 °C for 0 s); (4) cooling (1 cycle: 40 °C for 30 s). PCR products specificity was evaluated by melting curve analysis. Changes in expression levels were computed as mean fold changes, calculated using the comparative Ct method [59]. Δ*C*t was obtained by subtracting the mean *C*t of each sample to the mean *C*t of the reference gene under the exact experimental conditions. For the quantification of each gene we considered cDNAs from untreated RT4-D6P2T cells as the calibrator sample. The ΔΔ*C*t of each sample was then calculated by subtracting calibrator Δ*C*t to treated sample Δ*C*t. The formula 2^−ΔΔ*C*t^ was used to calculate fold changes. Baseline fold change for each calibrator sample were set to 1.

### 4.6. SDS-Polyacrylamide Gel Electrophoresis and Western Blotting

Western blot analysis was performed in lysates from RT4 cells following exposure to 1 µg/mL LPS for 24 h. Briefly, proteins were extracted using an ice-cold lysis buffer containing 20 mM Tris (pH 7.4), 2 mM EDTA, 0.5 mM EGTA; 50 mM mercaptoethanol, 0.32 mM sucrose, a protease inhibitor cocktail and the phosphatase inhibitor PhosSTOP (Roche Applied Science, Monza, Italy) using a Teflon-glass homogenizer and then sonicated twice for 20 s using an ultrasonic probe, followed by centrifugation at 10.000 *g* for 10 min at 4 °C. Protein concentrations were determined using the Quant-iT Protein Assay Kit (Invitrogen, Carlsbad, CA, USA). Sample proteins (30 μg) were diluted in 4× Laemmli buffer (Invitrogen), heated at 70 °C for 10 min and then separated on a BioRad Criterion XT 4–20% Tris-glycine gel (BioRad Laboratories) by electrophoresis and then transferred to PVDF membrane (Invitrogen). Non-specific sites were blocked with the Odyssey Blocking Buffer (Li-Cor Biosciences, Lincoln, NE, USA). Transfer was examined using a protein molecular weight marker (BioRad Laboratories). Western blot analyses were performed using the following primary rabbit polyclonal antibodies: PAC1 receptor (1:500, cat no# sc-30018, Santa Cruz Biotechnology Inc., Heidelberg, Germany), VPAC1 receptor (1:800, cat no# sc-30019, Santa Cruz Biotechnology Inc.), VPAC2 receptor (1:400, cat no# sc-30020, Santa Cruz Biotechnology Inc.), PACAP peptide (1:500, cat no# sc-25439, Santa Cruz Biotechnology Inc.), VIP peptide (1:1000, cat no# GTX129461, GeneTex) and GAPDH (1:2000, cat no# sc-47724, Santa Cruz Biotechnology Inc.). The secondary goat anti-rabbit antibody used was the IRDye 800CW, (cat #926-32211; Li-Cor Biosciences), which was used at the dilution of 1:20000. Blots were scanned using an Odyssey Infrared Imaging System (Li-Cor Biosciences) as detailed in previous work [60]. Densitometry was performed using the ImageJ software (NIH, Bethesda, MD; available at http://rsb.info.nih.gov/ij/). Optical densities of target proteins were normalized to GAPDH, which served as loading control. Background correction was also applied to reduce variability across membranes. 

### 4.7. MiRNA Profiling 

MiRNA concentrations and purity were determined by NanoDrop (ThermoFisher Scientific, Waltham, MA, USA). Only where miRNA extractions yielded ≥600 ng samples were used for downstream cDNA synthesis, which was obtained using the miScript II RT Kit (Qiagen). MiRNA profiling was then performed on cDNA templates using customised Rat miScript miRNA PCR arrays (384 well format from Qiagen) in a Roche LightCycler 480 instrument (Roche Diagnostics). Each cDNA was pre-mixed with the miScript Universal primer, QuantiTect SYBR Green PCR Master Mix and RNase-free water to a final volume of 10 µL before being added to the plates containing miRNA forward primers. The qPCR assay was set up as follows: 95 °C for 15 min; 40 cycles of 94 °C for 15 s; 55 °C for 30 s; and 70 °C for 30 s. The relative expression was calculated using the ΔΔ*C*t method as indicated above. Values were normalized to levels of the non-coding small nuclear RNA U6 (U6 snRNA). 

### 4.8. Statistical Analyses 

Statistical analysis was performed using GraphPad Prism^®^ version 7.02 software (GraphPad Software, Inc., La Jolla, CA, USA). Data were tested for normality with the Kolmogorov–Smirnov test. All variables were normally distributed. Unpaired Student’s t test was used for comparisons between two groups, whereas Dunnett’s *post-hoc* tests (unless otherwise indicated) were used to analyze whether groups’ data were significantly different following analysis of variance (between three or more treatment groups). The gene expression data collected from LPS treated samples at different time points were also analysed using linear regression to test for significant correlations between VIP/PACAP mRNA and select miRNA expression levels over time. Only where statistically significant correlations occurred are the data shown (Figure 7). All other correlations were not statistically significant. *P*-values of less than 0.05 (*p* < 0.05) were considered significant. All data are presented as the mean ± SEM and are the result of at least three independent determinations.

## Figures and Tables

**Table 1:** Primer sets used to determine mRNAs changes within the VIP/PACAP system in the RT4 Schwann cell line.

Gene (Ref. seq.)	Primers	Location of Primers	Tm (°C)	Length (bp)
Rattus norvegicus adenylate cyclase activating polypeptide 1 (*Adcyap1*) (NM_016989.2)	5′-GAGGCTTACGATCAGGACGG-3′	414	59.97	121
	3′-TCCTGTCGGCTGGGTAGTAA-5′	534	59.96	
Rattus norvegicus vasoactive intestinal peptide (*Vip*) (NM_053991.1)	5′-GTAGCATCTCGGAAGACCCC-3′	481	59.61	83
	3′-TTGCTTTCTAAGGCGGGTGT-5′	563	59.89	
Rattus norvegicus ADCYAP receptor type 1 (*Adcyap1r1*) (NM_001270579.1)	5′-GACCAGCATTCACCCCCTTT-3′	1442	60.25	114
	3′-CAGCCGTAGAGTAATGGTGGAT-5′	1555	59.63	
Rattus norvegicus vasoactive intestinal peptide receptor 1 (*Vipr1*) (NM_012685.2)	5′-AAGCTGCACTGTACCCGAAA-3′	597	59.89	103
	3′-CGCTGTTGAAGAGGGCCATA-5′	699	60.11	
Rattus norvegicus vasoactive intestinal peptide receptor 2 (*Vipr2*) (NM_017238.1)	5′-TGACCTGCTACTGCTGGTTG-3′	135	59.96	138
	3′-CGCTGCAAGCTCTGTGATTC-5′	272	59.9	
Rattus norvegicus ribosomal protein S18 (*Rps18*) (NM_213557.1)	5′-AGCGGCTGAAGAAAATCCGA-3′	380	60.04	115
	3′-TTGGACACACCCACAGTACG-5′	494	59.89	

Sense and antisense primers were selected from the 5′ and 3′ region of each gene coding region. The expected length of each amplicon is depicted in the right column.

**Table 2:** Selection of miRNAs based on their involvement in regulating biological processes relevant to the Schwann cell “repair phenotype”.

miRNA	Putative Role in SCs	Identified Targets	Reference(s)
miR-221	Cell migration, myelin genes	LAAS2	[18,19]
miR-222	Cell migration, myelin genes	LAAS2	[18,19]
**miR-29a**	Myelin genes, motility	PMP-22, CDK6	[20,21]
**miR-21**	Cell differentiation	SOX-2	[22]
miR-9	Cell migration	Cthrc1	[23]
**miR-27a**	Cell proliferation	FOXO1	[24]
**miR-34a**	De-differentiation genes, apoptosis	Notch1, Ccnd1, Bcl2, XLAP	[25,26]
**miR-132**	Cell migration	PRKAG3	[27]
**miR-145**	De-differentiation genes	Egr2, c-Jun, MPZ	[25,28]
miR-210	Cell proliferation & migration	GAP-43, MAG, MBP	[29]
**miR-146a**	Schwann cell development	SOX-10	[30]
**miR-155**	Inflammatory response	Toll-like receptors	[31]
**miR-181b**	Inflammation (in astrocytes)	MeCP2, XLAP	[32]
miR-182	Cell proliferation and migration	FGF9, NTM	[33]
**miR-204**	Apoptotic process	Neuritin	[34]
**miR-206**	Oncogenic transformation	NF2, ERBB2, NRG1	[35]
miR-137	Tumor suppressor function	NF1, MK2	[36,37]
**miR-340**	Production of proteolytic enzymes	tPA	[38]

MiRNAs whose expression levels were significantly dysregulated following LPS treatment are indicated in bold. Unaffected miRNAs are printed using regular font.
